# Causal effect of body mass index on herpes zoster and postherpetic neuralgia: A Mendelian randomization study

**DOI:** 10.1097/MD.0000000000042775

**Published:** 2025-06-06

**Authors:** Wen Wang, Ze Zhang, Qing Shi, Fuquan Wang, Yanting Cao, Bifa Fan, Yang Yang

**Affiliations:** a Department of Pain Management, China-Japan Friendship Hospital, Beijing, China; b Peking University China-Japan Friendship School of Clinical Medicine, Beijing, China; c Department of Graduate School, Beijing University of Chinese Medicine, Beijing, China; d Department of Anesthesiology, China-Japan Friendship Hospital, Beijing, China.

**Keywords:** body mass index, causality, herpes zoster, Mendelian randomization, postherpetic neuralgia

## Abstract

Although observational studies have reported the relationship between body mass index (BMI), herpes zoster (HZ), and postherpetic neuralgia (PHN), the impacts of BMI on the incidence of HZ and PHN are still controversial. Our study aimed to explore the causal effect of BMI on HZ and PHN by a 2‐sample Mendelian randomization (MR) approach. Genome-wide association studies data on BMI, HZ, and PHN were derived from publicly available genetic summary datasets. A total of 28 phenotypic single-nucleotide polymorphisms were selected as instrumental variables for BMI. Inverse-variance weighted (IVW) method was conducted as the primary MR analysis method to explore the causal effect of BMI on HZ and PHN. Several sensitivity analyses were performed to test the robustness of the MR results. Our study found no strong evidence for an effect of BMI on HZ incidence (IVW: OR = 1.018, 95% CI = 0.964–1.075, *P* = .524). However, it demonstrated that increased BMI was related to a higher risk of PHN (IVW: OR = 1.234, 95% CI = 1.002–1.520, *P* = .048). Besides, no significant heterogeneity or horizontal pleiotropy was observed in our study, and sensitivity analysis was consistent with the results. There is no causal effect of BMI on HZ risk, but it may be causally associated with a risk of PHN.

## 1. Introduction

Acute herpes zoster (HZ) results from the reactivation of varicella-zoster virus latent in the sensory ganglia. The incidence of HZ has been increasing annually with an individual lifetime risk of 25% to 30%.^[1,2]^ Postherpetic neuralgia (PHN) is the most common complication associated with HZ, defining as pain persisting more than 90 days after HZ. PHN occurring in approximately 40% of patients is persistent chronic pain, which is difficult to manage and greatly influences quality of life.^[3,4]^ Thus, identifying the potential risk factor is of great significance for the prevention and treatment of HZ and PHN.

Body mass index (BMI) is an index of the degree of obesity and health. Numerous observational studies have been conducted to explore the effect of BMI on HZ and PHN. However, whether high BMI has a protective or risk effect on the incidence of HZ and PHN is still controversial.^[5–7]^

Mendelian randomization (MR) is an epidemiological method which explore the impact of risk factors on disease outcomes by selecting variants as instrumental variables (IVs) from genome-wide association studies (GWAS).^[8]^ This method can effectively overcome potential confounding and reverse causation,^[9]^ and it has been increasingly used as a valuable complement to traditional observational studies for causal inference of etiology.

Thus, we conducted a 2-sample MR analysis aiming to reveal the causal effect of BMI on HZ and PHN.

## 2. Methods

### 2.1. Study design

A 2-sample MR study was conducted to explore the causal effect of BMI on HZ and PHN. In MR study, representative phenotypic single-nucleotide polymorphisms (SNPs) directly associated with BMI were chosen as genetic IVs. To obtain unbiased results, IVs should meet the following 3 main assumptions^[9]^: (1) IVs must be strongly associated with exposure factors (the relevance assumption); (2) IVs should be independent of any potential confounders of the exposure-outcome association (the independent assumption); (3) IVs must be correlated with outcomes only through exposure factor but not any other causal pathway (the exclusion restriction assumption).

Our study was published according to the MR-STROBE recommendation.^[10]^ Ethical approval was exempt from the Ethical Review Authority because the data analyzed in this study were anonymized, deidentified, and publicly available.

### 2.2. Data source

GWAS summary-level data used in our study were obtained from 2 public datasets including the Integrative Epidemiology Unit (IEU) open GWAS project (https://gwas.mrcieu.ac.uk) and the FinnGen Release 11, (https://www.finngen.fi/en).

The GWAS data for BMI were obtained directly from IEU open GWAS project (https://gwas.mrcieu.ac.uk), deposited under ID ieu-b-4816 containing 99,998 European-descent individuals and 7191,606 SNP sites related to BMI.^[11]^ Developed at the MRC IEU at the University of Bristol, this project is a manually curated collection of complete GWAS summary datasets made available as open-source files for download, or by querying a database of the complete data.

To ensure analytical accuracy and avoid sample size overlap, the GWAS data for HZ and PHN came from the FinnGen Release 11, published on June 24, 2024 (https://www.finngen.fi/en). The project is a collaboration between research organizations and biobanks within Finland and international industry partners. It is a large-scale genomics initiative that has analyzed over 500,000 Finnish biobank samples and correlated genetic variation with health data to understand disease mechanisms and predispositions. The total number of European-descent participants for HZ was 442,140 (including 6268 cases and 435,872 controls). For PHN, the total number of participants was 396,252 (including 420 cases and 395,832 controls).^[12]^

Detailed information about these 3 GWAS datasets is shown in Table 1.

**Table 1:** GWAS datasets for BMI, HZ, and PHN.

	BMI	HZ	PHN
Source	IEU open GWAS project (https://gwas.mrcieu.ac.uk)	FinnGen biobank (https://www.finngen.fi/en)	FinnGen biobank (https://www.finngen.fi/en)
Population	European	European	European
Sex	Males and Females	Males and Females	Males and Females
Ncase	NA	6268	420
Ncontrol	NA	435,872	395,832
Total sample size	99,998	442,140	396,252
Author	Howe LJ	NA	NA
GWAS ID	ieu-b-4816	NA	NA
PMID	NA	NA	NA

BMI = body mass index, GWAS = genome-wide association studies, HZ = herpes zoster, NA = not available, Ncase = number of cases, Ncontrol = number of controls, PHN = postherpetic neuralgia.

### 2.3. SNPs selection

Briefly, BMI served as the exposure, whereas HZ and PHN served as the outcomes. The SNPs were screened following several selection criteria: (1) Eligible SNPs in the exposed dataset were identified as IVs for BMI at a significance level of *P* < 5 × 10^−8^. (2) The SNPs exhibiting negligible linkage disequilibrium effects were excluded with *R*^2^ > 0.001 and distance = 10,000 kb apart from each other. (3) *F* statistic (*F* = beta^2^/se^2^) for each IV was calculated to assess the strength of IV. To avoid potential weak instrumental bias, only those IVs with *F* > 10 were retained.^[13]^ (4) Data on exposure and outcome were merged and harmonized according to effect alleles, and SNPs that were potentially correlated with the outcome (*P* < 5 × 10^−8^) were discarded in the merged data. (5) These SNPs were searched and checked in the GWAS Catalog (https://www.ebi.ac.uk/gwas) to eliminate those confounding variables linked to the exposure-outcome association.^[14]^

### 2.4. Mendelian randomization and visualizations

To analyze the causal effect of BMI on HZ and PHN, 5 popular MR methods were applied including inverse-variance weighted (IVW), weighted median, MR-Egger (ME), simple mode, and weighted mode.^[15–18]^ In our MR study, IVW was the main statistical method to test the causal relationship, and the other 4 served as complements for the former is reported to be slightly more powerful than the others under certain conditions.^[19]^ The results of the MR analysis were shown visually via funnel plots, and scatter plots, and leave-one-out plots.

### 2.5. Sensitivity analysis

To ensure the validity and robustness of MR results, several sensitivity analyses were performed. Heterogeneity of effects shows the variability in the causal estimates and pleiotropy potentially indicates that other exposure variant affects the outcome. Thus, it is very necessary to apply heterogeneity and pleiotropy testing in MR analyses. Significance of heterogeneity or pleiotropy testing means that potential biases exist in causal estimates and unreliable results are obtained. In our study, heterogeneity was evaluated by the Cochran Q test using IVW and ME methods with *P* < .05 indicating heterogeneity.^[20,21]^ Besides, ME intercept analysis was employed to check horizontal pleiotropy.^[22]^ If the intercept term was significant (*P* < .05), the existence of horizontal pleiotropy was indicated. Leave-one-out analysis was then further employed by removing every SNP one by one to test the consistency of the results.^[23]^

### 2.6. Statistical analysis

All the above statistical procedures were performed using the TwoSampleMR package (version 0.6.6) in the Rstudio (R version 4.4.1). The causal relationships between BMI and outcomes were expressed as beta(β)/odds ratios (ORs) and 95% confidence interval (CI). Threshold of significance was set as *P* < .05.

## 3. Results

The following results were obtained after the above methods.

### 3.1. IVs Selection for BMI

After screening, 42 SNPs intensively associated with BMI were extracted as initial IVs (see Supplemental file 1, Supplemental Digital Content, https://links.lww.com/MD/P138). Then, 7 SNPs (rs10055107, rs12142020, rs2140664, rs4670454, rs59778458, rs72705208, and rs9788550) were removed for being palindromic with intermediate allele frequencies after harmonizing exposure and outcome. Next, another 7 SNPs (rs12140153, rs13130484, rs633715, rs7132908, rs8050136, rs8089364, and rs9568868) were eliminated as they were linked to well-established risk elements for HZ and PHN such as diabetes.^[24,25]^ Finally, 28 SNPs were identified to assess the causal effect of BMI on HZ and PHN. Two sets of 28 IVs for both HZ and PHN were the same and they were strong with *F* values all >10 (range: 30–155). Thus, there was no bias caused by weak IVs in our study (see Supplemental file 2,Supplemental Digital Content, https://links.lww.com/MD/P139).

### 3.2. Causal effect of BMI on HZ

**Figure 1.**
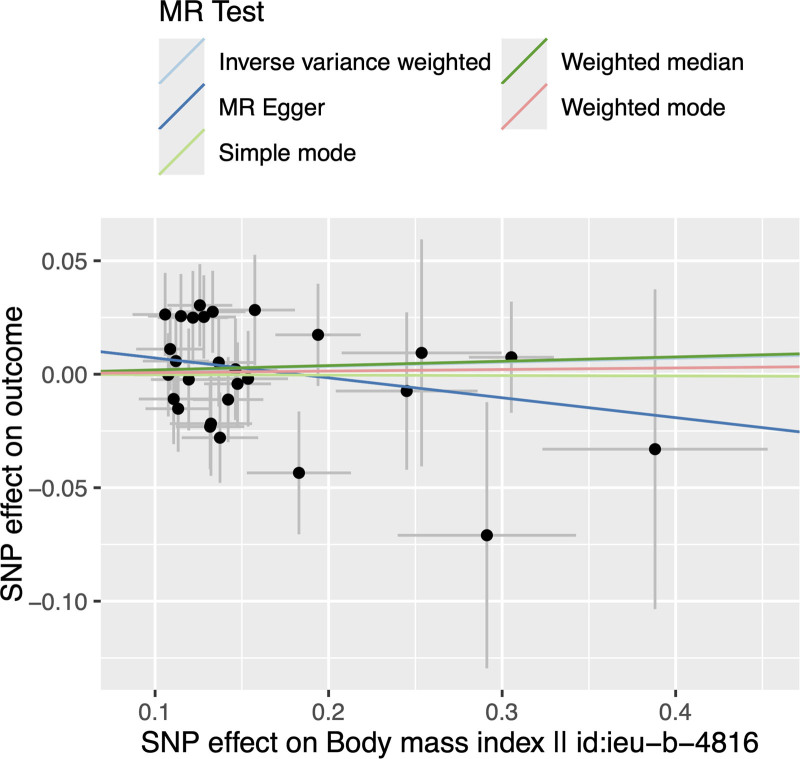
Scatter plots showing the causal effect of body mass index (BMI) on herpes zoster (HZ).

**Figure 2.**
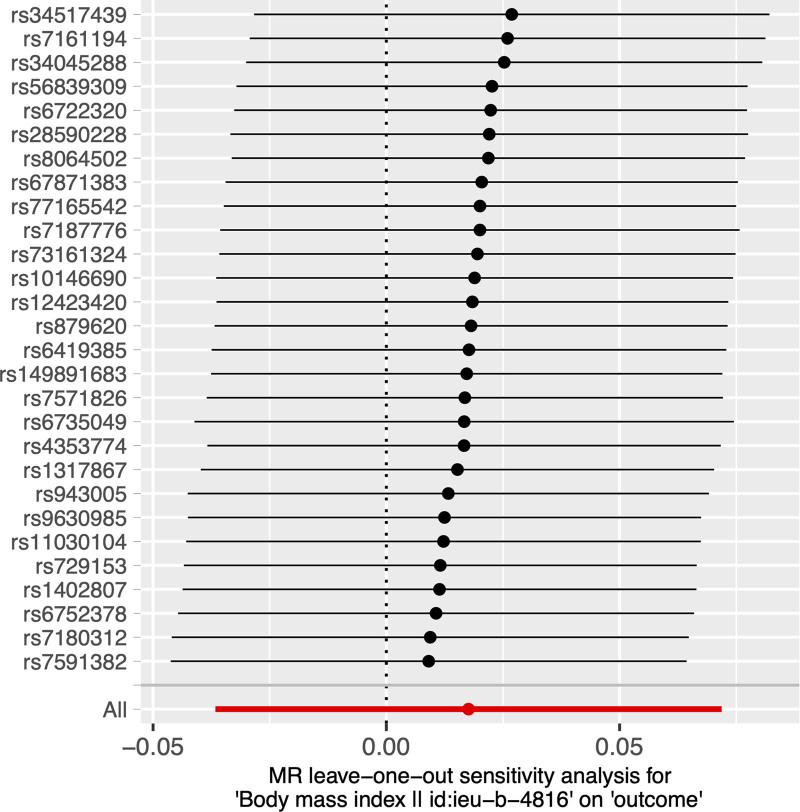
Leave-one-out analysis for the impact of individual SNPs on the association between body mass index (BMI) and herpes zoster (HZ) risk.

Our study found no causal effect of BMI on HZ (IVW, OR = 1.018, 95% CI = 0.964–1.075, *P* = 0. 524). The results of the other 4 methods yielded similar results and details are shown in Table 2. In addition, no significant heterogeneity was found in the MR analysis (ME Q = 23.407, *P* = .610; IVW Q = 24.732, *P* = .590; Table 3). The ME intercept is close to 0 with *P* > .05 (Egger-intercept = 0.016; *P* = .260), suggesting no horizontal pleiotropy (Table 4). The scatter plot is shown in Figure 1. By leaving out exactly 1 SNP, the leave-one-out analysis showed that the causal effect of BMI on HZ was not influenced by individual SNP (Fig. 2).

**Table 2:** Two-sample MR analysis of causal effect of BMI on HZ and PHN.

Exposure	Outcome	Method	nSNP	Beta	SE	OR (95% CI)	*P*
BMI	HZ	IVW	28	0.018	0.028	1.018 (0.964–1.075)	.524
		ME		−0.088	0.096	0.916 (0.759–1.105)	.367
		WMn		0.019	0.041	1.019 (0.941–1.104)	.641
		SM		−0.002	0.082	0.998 (0.850–1.172)	.982
		WMd		0.007	0.066	1.007 (0.886–1.145)	.916
BMI	PHN	IVW	28	0.210	0.107	1.234 (1.002–1.520)	.048
		ME		0.022	0.373	1.022 (0.492–2.124)	.954
		WMn		0.123	0.150	1.130 (0.842–1.517)	.414
		SM		0.125	0.277	1.134 (0.658–1.952)	.655
		WMd		0.117	0.219	1.124 (0.733–1.725)	.596

BMI = body mass index, CI = confidence interval, HZ = herpes zoster, IVW = inverse variance weighted, ME = MR-Egger, MR = Mendelian randomization, OR = odds ratio, PHN = postherpetic neuralgia, SE = standard error, SM = simple mode, SNP = single-nucleotide polymorphism, WMd = weighted mode, WMn = weighted median.

**Table 3:** Heterogeneity and pleiotropy tests in MR analysis.

Exposure	Outcome	Method	Cochran’s Q	Q_df	*P*
BMI	HZ	ME	23.407	26	.610
		IVW	24.732	27	.590
BMI	PHN	ME	26.960	26	.411
		IVW	27.248	27	.450

BMI = body mass index, HZ = herpes zoster, IVW = inverse variance weighted, ME = MR-Egger, MR = Mendelian randomization, PHN = postherpetic neuralgia.

**Table 4:** Pleiotropy tests in MR analysis.

Exposure	Outcome	Egger-intercept	SE	*P*
BMI	HZ	0.016	0.014	.260
BMI	PHN	0.029	0.054	.602

BMI = body mass index, HZ = herpes zoster, ME = MR-Egger, MR = Mendelian randomization, PHN = postherpetic neuralgia, SE = standard error.

### 3.3. Causal effect of BMI on PHN

**Figure 3.**
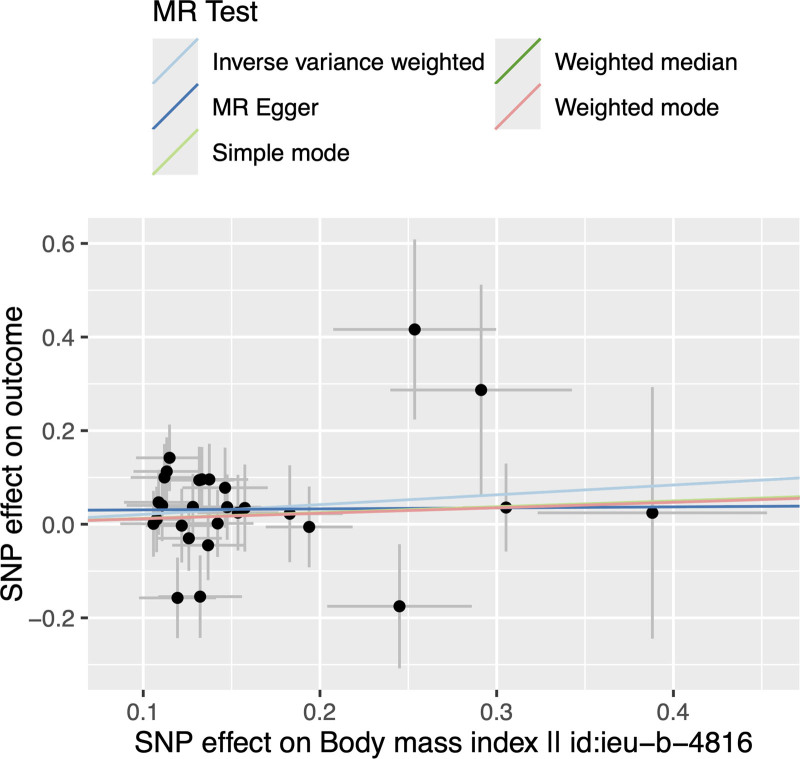
Scatter plots showing the causal effect of body mass index (BMI) on postherpetic neuralgia (PHN).

**Figure 4.**
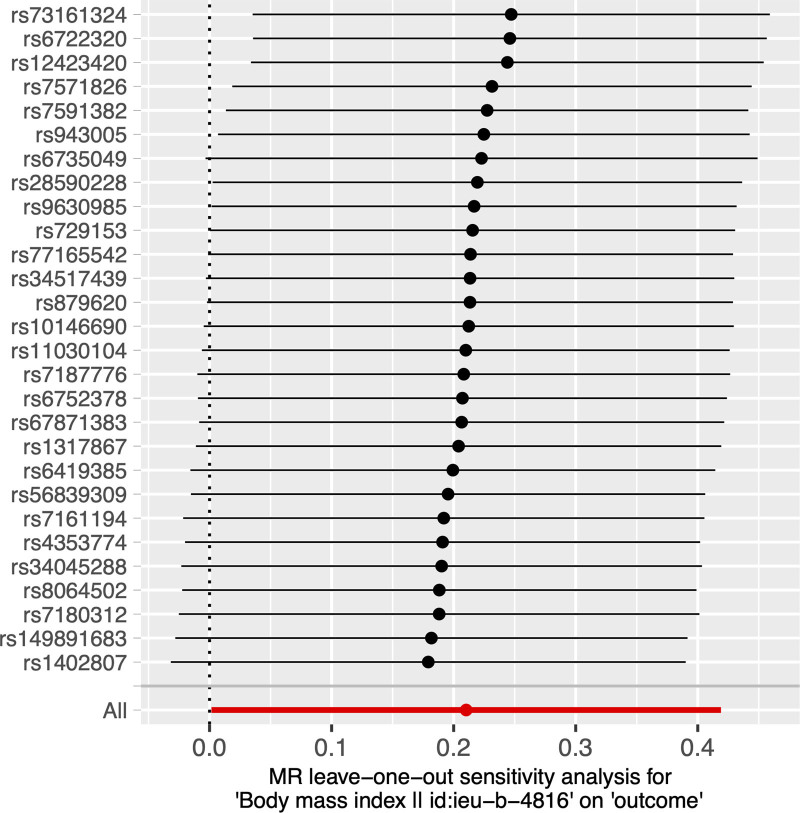
Leave-one-out analysis for the impact of individual SNPs on the association between body mass index (BMI) and postherpetic neuralgia (PHN) risk.

In the MR analysis of the causal effect of BMI on PHN, a *P* value below .05 was observed according to IVW (OR = 1.234, 95% CI = 1.002–1.520, *P* = .048), without significant heterogeneity (ME Q = 26.960, *P* = .411; IVW Q = 27.248, *P* = .450; Table 3) and pleiotropy (Egger-intercept = 0.029, *P* = .602; Table 4). The results of the causal effect using the other 4 methods are shown in Table 2. The scatter plot is shown in Figure 3. Besides, the leave-one-out analysis showed that the causal effect of BMI on PHN was not influenced by individual SNP (Fig. 4).

## 4. Discussion

Our results demonstrate that genetically predicted BMI has no causal effect on the risk of HZ, but it may be causally associated with a risk of PHN. The above findings were obtained by applying a 2‐sample MR analysis to assess whether BMI causally affects HZ or PHN incidence and they have been considered robust when validated by a series of sensitivity analyses.

Pain is one of the most common complications associated with HZ, including acute herpetic pain and PHN.^[26]^ Both types of pain can seriously affect the quality of patients’ life.^[27]^ Thus, recognizing risk factor is important for the prevention and treatment of HZ and PHN. Although many researchers have reported the relationship between BMI, HZ, and PHN, their results have still been inconsistent. Some studies have indicated a positive correlation between BMI and HZ, with higher BMI related to a lower incidence of HZ.^[7,28]^ However, others have observed that both underweight and obese can lead to increased risk of PHN.^[29]^ Moreover, there are also studies demonstrating that the impact of BMI on the incidence of HZ or PHN is unclear.^[5,6]^

Inconsistent results between these studies may be due to the inconsistency of study design, study population, sample size, inclusion criteria, definition of HZ and PHN although all these studies were clinical.^[5–7,28,29]^

Our study is the first to employ MR method to systematically investigate the causal relationship among BMI, HZ, and PHN within a European population, which could reveal causality due to better study design. Compared with observational studies, MR is a statistical method that can effectively decrease the influence of confounding factors by selecting genetic variants as IVs for exposure assessment from GWAS rather than individual-level data.^[30,31]^

In our study, we adopted several ways to improve the efficiency of MR analysis. Large samples of GWAS data on exposure and outcomes were obtained from individuals of European ancestry. Moreover, diabetes is confounding factor when analyzing the association between BMI, HZ, and PHN as it was one of well-established risk factors for HZ/PHN and other problems according to the existing studies.^[32–34]^ Thus, 7 SNPs closely related to HZ and PHN were removed from our study. Besides, more power IVs, more statistical methods, and heterogeneity-testing methods were included to assess the possibility of pleiotropy, prevent potential bias, and improve the robustness of the results. Possible biological mechanisms of BMI affecting the occurrence of PHN may be through immune response. Previous studies have confirmed the proinflammatory status of obese individuals and demonstrated a positive correlation between BMI and a proinflammatory poststroke immune response.^[35,36]^ A recent study has shown that persistent or altered activation of nonspecific immunity may contribute to the development of PHN from HZ.^[37]^

However, there are still some limitations in our study that should be noted when interpreting our findings. First, the findings should be generalized carefully as all GWAS data came from databases and European population although MR analysis was applied. Second, attention should be paid to the highly polygenic trait of BMI with heritability estimated to be between 30% and 40%.^[38]^ Recent studies have found that complex traits such as height, BMI are affected by a large amount of genetic variants of small effects.^[39,40]^ Genome-wide significant SNPs often explain only a small proportion of heritability.^[41]^ Besides, it is also influenced by various environmental and behavioral risk factors.^[42]^ Thus, pleiotropy may not be completely excluded from our study. Third, stratification analysis for BMI was not feasible because of the absence of more detailed clinical information of the study participants, which may lead to the “obesity paradox” due to extreme BMI.^[43]^ The obesity paradox means available evidence demonstrating that obesity in patients with chronic diseases may be protective and associated with decreased mortality.^[44]^

## 5. Conclusions

Our study provided evidence that higher BMI may be causally associated with an increased risk of PHN but did not support a causal relationship between BMI and HZ. Thus, keeping BMI at a certain level is important for the clinical prevention of PHN in public health. However, we do not know yet how different levels of BMI influence HZ and PHN, or whether certain BMI ranges have a greater impact on risk. Further research including subgroups of BMI should be prompted in the future.

## Acknowledgments

Genetic association data for body mass index, herpes zoster, and postherpetic neuralgia were obtained from IEU open GWAS project database and FinnGen Consortium. The authors would like to thank the participants and researchers of IEU open GWAS project database and FinnGen Consortium for providing relevant SNP and GWAS data.

## Author contributions

**Conceptualization:** Wen Wang, Yang Yang.

**Writing – original draft:** Wen Wang.

**Data curation:** Ze Zhang, Qing Shi, Yanting Cao, Fuquan Wang.

**Funding acquisition:** Bifa Fan, Yang Yang.

**Supervision:** Bifa Fan.

**Writing – review & editing:** Yang Yang.

## Supplementary Material


